# Notes on the Spreading of *Penaeus aztecus* Ives 1891 (Decapoda, Penaeidae) in the Mediterranean Sea and on Its Repeated Misidentifications in the Region

**DOI:** 10.3390/biology12060793

**Published:** 2023-05-30

**Authors:** Carlo Froglia, Martina Scanu

**Affiliations:** 1 CNR—Institute for Biological Resources and Marine Biotechnologies (IRBIM), Largo Fiera della Pesca 2, 60125 Ancona, Italy; martina.scanu@irbim.cnr.it; 2 Department of Biological, Geological, and Environmental Sciences (BiGeA), Alma Mater Studiorum—University of Bologna, Piazza di Porta S. Donato 1, 40126 Bologna, Italy

**Keywords:** Crustacea Decapoda, *Penaeus aztecus*, non-indigenous species, vector, Adriatic Sea, Mediterranean Sea, Black Sea

## Abstract

**Simple Summary:**

The shrimp *Penaeus aztecus*, native to the western Atlantic, was first reported in the Mediterranean Sea (Bay of Antalya, Southern Turkey) in 2010. In the following years, it proved its invasiveness with multiple records from all over the Mediterranean except the westernmost sector and the North Adriatic Sea. Several pieces of evidence suggest that the unintentional transport of larvae in the ballast waters of transoceanic vessels departing from the U.S. West Coast, instead of the escape of adults from unreported experimental shrimp farming, is the more likely pathway of the introduction of *P. aztecus* in the Mediterranean Sea. The accurate scrutiny of scientific literature on non-indigenous species brought to light an earlier (2005) arrival in the Black Sea, which passed unnoticed as the shrimps were misidentified as *Penaeus semisulcatus*, also a non-indigenous species, which is established and exploited in the Levant Sea since 90 years. But it is native to the Indo-Pacific region, other misidentifications were also found, therefore morphological characters allowing correct identification of the two species and of the autochthonous *Penaeus kerathurus* are illustrated. Non-indigenous species are among the descriptors adopted in the Marine Strategy Framework Directive for determining the good environmental status of marine waters in the European States, hence the importance of their correct identification.

**Abstract:**

The shrimp *Penaeus aztecus*, native to the western Atlantic, was first reported in the eastern Mediterranean Sea in 2010. New records, from different Mediterranean localities, multiplied in the following years. The accurate search of the literature on non-indigenous species discovered it was misidentified more than once as another alien shrimp, *P. semisulcatus*, native to the Indo-Pacific region, with the result that its earlier presence in the Black Sea went unnoticed. Morphological characteristics allowing the identification of these two species, the autochthonous *P. kerathurus* and two other alien *Penaeus* species present in the Mediterranean, are reprised. The present distribution of *P. aztecus* based on literature records and surveys carried out in the northern and central Adriatic between 2016 and 2021 is mapped. The unintentional transport of larvae carried in ballast water by transoceanic vessels departing from the U.S. East Coast is suggested as the most probable introduction pathway. The significance of the correct identification of non-indigenous species, a “Descriptor” adopted in the Marine Strategy Framework Directive for determining the good environmental status of marine waters in the European States, is emphasized.

## 1. Introduction

The unintentional introduction of non-indigenous species (NIS) in marine habitats as a consequence of maritime activities is a worldwide phenomenon. The evolution of naval architecture, with the adoption of tanks for the temporary storage of large volumes of seawater to ballast unloaded cargo vessels together with the progressive increase of vessel speed, gave new opportunities to plankton and larval stages of benthic species to be displaced outside their native range and to settle in new habitats [1]. Ship canals, opened to facilitate maritime traffics, also facilitate the introduction of NIS. The Suez Canal, progressively enlarged since its opening in 1869 [2], has reconnected the Atlanto-Mediterranean and the Indian-Red Sea biota, which have been separated for several million years. A large number of Red Sea immigrants, “Lessepsian immigrants” [3], have entered the Mediterranean through this waterway and have progressively changed the biota of the eastern Mediterranean coastal waters. Substantial differences between the eastern and western Mediterranean are observed in the total number of NIS, their native regions, and pathways of introduction [4,5].

The “brown shrimp” *Penaeus aztecus* Ives, 1891 is native to the NW Atlantic and Gulf of Mexico, where it is a very important fishery resource, with annual landings of over 40,000 tons [6]. Its presence in the Mediterranean Sea was reported for the first time in 2010 [7] from several specimens collected in the Gulf of Antalya (South Turkey) since December 2009. The unintentional transport of larvae via ballast waters was suggested as the most probable vector of introduction [7]. Only 3 years later, *P. aztecus* became common, not only in the coastal waters of southern Turkey [8], but also in the northern Aegean Sea (Thermaikos Gulf) [9]. A single specimen was also caught in the South Adriatic (Boka Kotorska) [10]. In the following years, the capture of one, or a few specimens, was reported from several localities all over the Mediterranean Sea and the hypothesis of escapes from aquaculture plants was also suggested [11,12,13].

To follow the chronology of the spreading of *P. aztecus* in the Mediterranean and its present distribution, an in-depth scrutiny of the literature was carried out. It also discovered the species was misidentified as *Penaeus semisulcatus* de Haan, 1844, in the Black Sea [14,15] and Central Mediterranean [16].

In this age of globalization, species native from all over the world can easily reach our shores. Therefore, it is fundamental that the new records of alien species include detailed illustrations of the specimens examined. It allows taxonomists to detect possible misidentifications, otherwise perpetuated by their inclusion in regional lists of alien species, as in the herein-discussed cases of *P. aztecus.*

## 2. Materials and Methods

We suspected that the report of *Penaeus semisulcatus* from the Gulf of Taranto (West Ionian Sea) published in 2015 [16] was the result of a misidentification of *P. aztecus*; therefore, in the summer of 2016, a leaflet with photos of distinctive characters of the shrimps was produced and sent to a friend, a skilled artisanal fisher in Roccella Ionica (Ionian Sea). Quite soon, we received photos and the first specimen caught with a trammel net. At the same time, skippers of bottom trawlers in Ancona (Central Adriatic Sea) told us that occasionally they noted single “Mazzancolle”—the Italian commercial name for the autochthonous *P. kerathurus* (Forskål, 1775)—with uniform color, without the typical dark transversal bands. Therefore, in October 2016, the same leaflet was circulated in the wholesale fish markets of Ancona and San Benedetto del Tronto, the largest in the Central Adriatic, where large quantities of “Mazzancolle” are auctioned daily. Shortly after, we received shrimp specimens from both markets, with the indication of the fishing area where they were caught.

All shrimps received were preserved in 80% ethanol and stored in the CF Decapoda collection, which is to be transferred to the Museo Civico di Storia Naturale in Verona, Italy.

We also received various reports from fishers, but not supported by physical specimens; these are not included here.

The number of records of an NIS may indicate its dispersal capability, but they are a poor index of its actual abundance. To obtain insight into the relative abundance of *P. aztecus* versus the autochthonous *P. kerathurus,* here we examine the data collected between 2016 and 2021 during the fishery surveys “SoleMon”. It is carried out in the Northern and Central Adriatic (GSA 17), to assess the abundance of some flatfish stock as well as commercial invertebrates [17]. This survey is carried out yearly in late autumn, when juveniles of common sole, common cuttlefish, and shrimp have left their nursery areas (lagoons and coastal waters) and are recruited to the fishery. In each survey, about 70 stations—located between the Italian and Slovenian coast and the limit of Croatian territorial waters, depth range 10–80 m—have been sampled with two “rapido”, professional beam trawls (width 3.6 m) rigged with iron teeth along the lower leading edge [17] (Annex 1), towed for 30 min at an average speed of 5.5 knots (about 10 km/h). In each sampling station, penaeid shrimps have been identified to species, sexed, counted, and measured.

The review of the literature and databases on NIS, present on the Web, brought to light other erroneous reports. Therefore, to facilitate the identification of *P. aztecus* from *P. semisulcatus*, the autochthonous *P. kerathurus,* and two other alien *Penaeus* species present in the Mediterranean Sea (namely *P. pulchricaudatus* Stebbing, 1914 and *P. hator* Burkenroad, 1959, both native to the Indian Ocean), more evident distinctive characters are summarized and illustrated. 

## 3. Results

### 3.1. Species Identification

In the earlier records from the Mediterranean Sea, the shrimp species herein considered has been reported under the name *Farfantepenaeus aztecus* (Ives, 1891), following the nomenclature adopted by Pérez-Farfante and Kensley [18]. They split the genus *Penaeus* Fabricius, 1798 into six genera, based on morphological differences. In a phylogenetic molecular study, published in 2011 [19], these genera were again lumped into the genus *Penaeus* s.l.; thereafter, the species was reported under the name *Penaeus aztecus* Ives, 1891. In a very recent comprehensive phylogenetic molecular investigation, Yang et al. [20], while keeping all the species in the genus *Penaeus* s.l., showed that up to 11 clades can be recognized within the genus. Chan [21] morphologically characterized these clades, regarded as subgenera of *Penaeus* s.l., and reinstated *Farfantepenaeus* Burukovsky, 1972 at the subgenus level. In this note, we use the genus name *Penaeus* for all the species considered. 

The 13 shrimps examined have been identified as adult *Penaeus aztecus* based on a set of morphological characters reported in the literature [22] and the comparison with the material of *P. aztecus, P. semisulcatus, P. hator, P. pulchricaudatus,* and *P. kerathurus* present in the Decapoda collection of the senior author (CF).

Material examined, *Penaeus aztecus*:

Western Ionian Sea: 1♂ c.l. 30 mm, off Roccella Ionica, depth 10 m, 30 July 2016, trammel net, D1962; 2♀ c.l. 36–41 mm, same locality, July 2017, D1961.

Central Adriatic Sea: 4♀ c.l. 41.3–44.5 mm, Ancona, about 6 miles (11 km) offshore, depth 30–35 m, October 2016, coastal trawlers, D1959; 1♀ c.l. 43 mm, S. Benedetto del Tronto, about 6 miles (11 km) offshore, depth 30 m, 11 November 2016, coastal trawler, D1960; 1♂ c.l. 29.3 mm, S. Benedetto del Tronto, about 3 miles (5.5 km) offshore, depth 15 m, 30 July 2017, beam trawler, D2136; 2♀ c.l. 48–53 mm, off Porto Civitanova, 13 September 2017, coastal trawler, D2137; 1♀ c.l. 40 mm, 43°20′ N 13°59′ E (SoleMon 2020 St. 67), depth 52–53 m, 15 December 2020, D2138; 1♀ c.l. 40 mm, 43°42.4′ N 13°41.9′ E (SoleMon 2021 St. 36), depth 52–53 m, 28 November 2021, D2139.

Comparative material:

*Penaeus aztecus*: 1♂ c.l. 10.5 mm, 2♀ c.l. 10.0–11.8 mm, 29°30′ N 91°52′ W, Missisipi Delta, USA, depth 2 m, 30 May 1999, D1775; 2♂, 1♀, 36°49′ N 30°58′ E, Gulf of Antalya East Mediterranean, depth 30 m, 26 June 2010, D2100.

*Penaeus semisulcatus*: 1♂ c.l. 35.6 mm, 1♀ c.l. 43.2 mm, 31°15′ N 32°41′ E, Israel, East Mediterranean, depth 16 m, 31 October 1975, D255; 3♂ c.l. 30.8–42.1 mm, 1♀ c.l. 48.5 mm, 36°47′ N 31°15′ E, Gulf of Antalya, East Mediterranean, depth 40 m, 22 June 2010, D2153.

*Penaeus kerathurus*: 2♂ c.l. 24.6–30.2 mm, 2♀ c.l. 21.3–31.8 mm, 43°45′ N 13°18′ E, Central Adriatic, depth 14 m, 17 December 1984, D1313; 1♂ c.l. 39.3 mm, 1♀ c.l. 48 mm, 43°06.1′ N 13°54.3′ E, Central Adriatic, depth 12 m, 29 August 2001, D2031.

*Penaeus hator*: 1♂ c.l. 37.9 mm, 1♀ c.l. 39.0 mm, 36°49′ N 31°02′ E, Gulf of Antalya, East Mediterranean, depth 30 m, 21 April 2011, D2155.

*Penaeus pulchricaudatus*: 1♂ c.l. 43 mm, 1♀ c.l. 56.5 mm, off Bardawil lagoon, East Mediterranean, depth 18 m, 27 January 1979, D1931.

 

**Figure 1:**
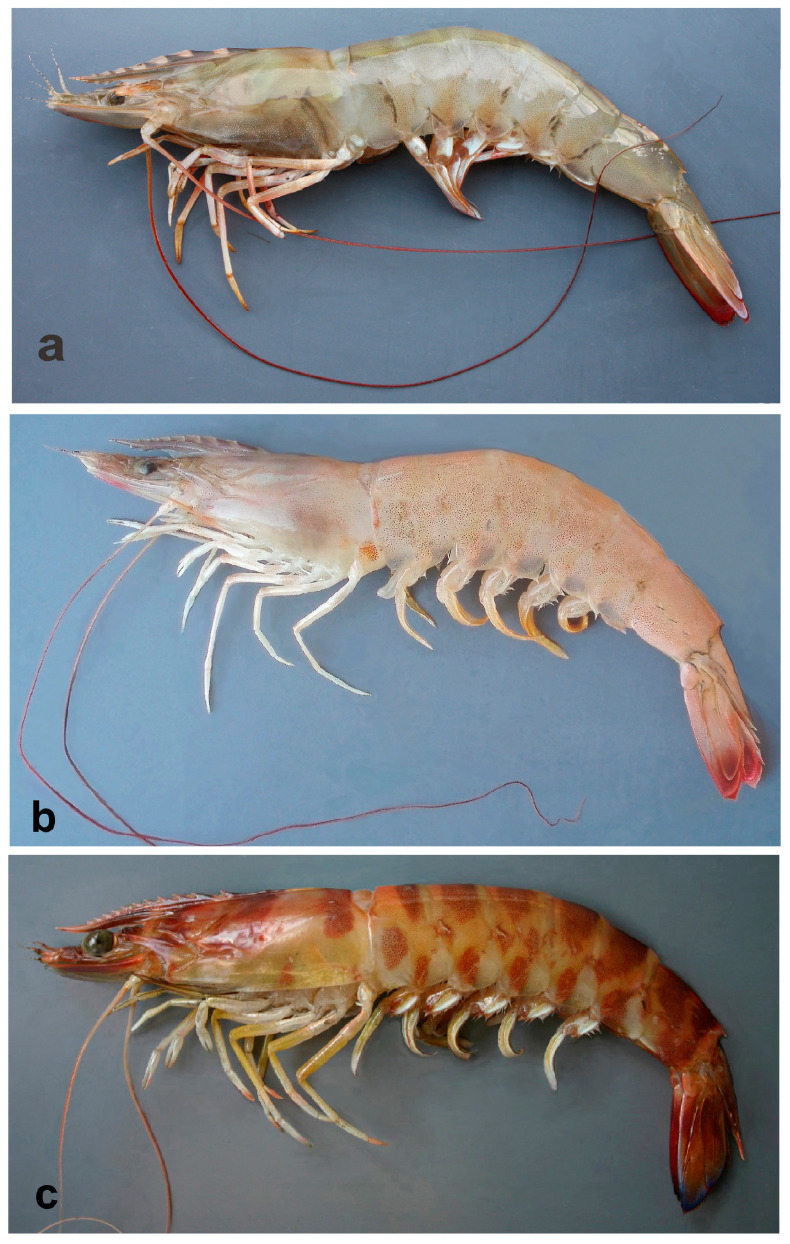
Colors in life: (**a**) *Penaeus semisulcatus*, eastern Mediterranean Gulf of Antalya; (**b**) *Penaeus aztecus*, Adriatic Sea off Ancona; (**c**) *Penaeus kerathurus*, Adriatic Sea off Ancona.

In freshly caught *P. aztecus,* the body color is light brown to rose with minute reddish chromatophores, and uropods have reddish distal margins, whereas *P. semisulcatus* is olive-brown, with slightly darker transverse bands and reddish setal fringe of uropods, and *P. kerathurus* is light brown with dark brown interrupted transverse bands (may fade after long storage in ice, but always remain visible), the uropods are distally bluish (Figure 1). The color pattern in *P. pulchricaudatus* is similar to that of *P. kerathurus*, except for the uninterrupted dark brown bands on abdominal somites. *P. hator* presents a cream color body with short narrow vertical brown stripes on abdominal pleurae.

**Figure 2:**
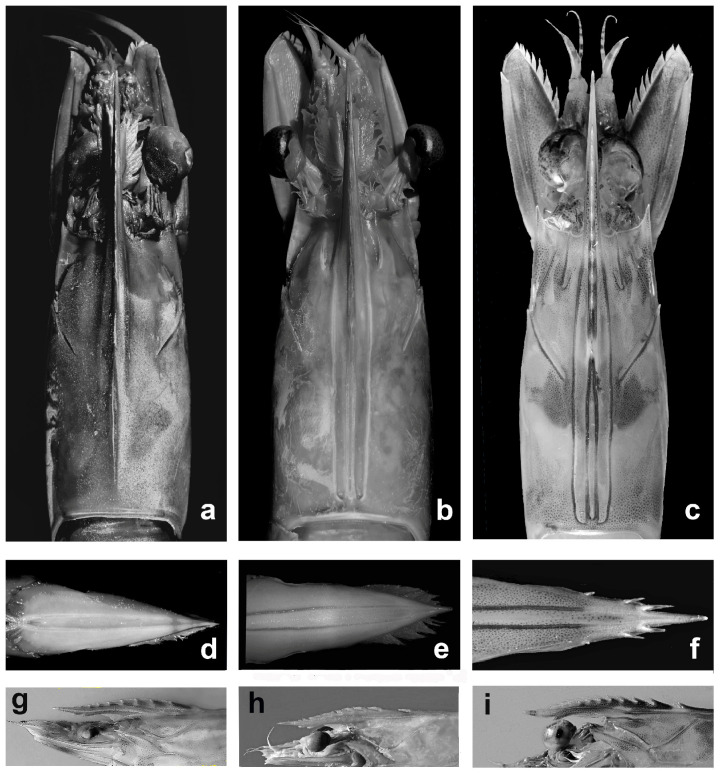
*Penaeus semisulcatus* (**a**,**d**,**g**); *Penaeus aztecus* (**b**,**e**,**h**); *Penaeus kerathurus* (**c**,**f**,**i**); carapace dorsal view (**a**–**c**); telson (**d**–**f**); rostrum side view (**g**–**i**).

Preserved specimens, with colors faded, can be easily identified on a set of morphological characters (Figure 2):

- The adrostral groove and crest end about at 2/3 of the carapace length in *P. semisulcatus,* whereas extend almost to the posterior margin of the carapace in *P. aztecus* and *P. kerathurus*, as well as in *P. pulchricaudatus* and *P. hator*;
- The ventral margin of the rostrum bears two, occasionally three, teeth in *P. aztecus*, versus three to four in *P. semisulcatus,* and only one in *P. kerathurus, P. pulchricaudatus,* and *P. hator*;
- The lateral margins of telson are devoid of teeth or spines in *P. aztecus* and *P. semisulcatus,* whereas are distally armed with three pairs of movable spines in *P. kerathurus*, *P. pulchricaudatus,* and *P. hator*;
- The mesial margin of both coxa and basis of the first and second pereopods are armed with acute teeth in *P. kerathurus,* whereas the other four species lack coxal teeth on the first and second pereopods; only *P. aztecus* has an ischial tooth on the first pereopods;
- The last abdominal somite in *P. aztecus* presents a well-defined dorsolateral sulcus, lacking in the other four species.

### 3.2. Previous Misidentifications

The accurate scrutiny of the existing literature on Mediterranean NIS, primarily carried out to investigate the spreading of *P. aztecus*, discovered that it has been repeatedly misidentified as *P. semisulcatus* de Haan, 1844, an NIS native to the Red Sea and the Indian Ocean.

**Figure 3:**
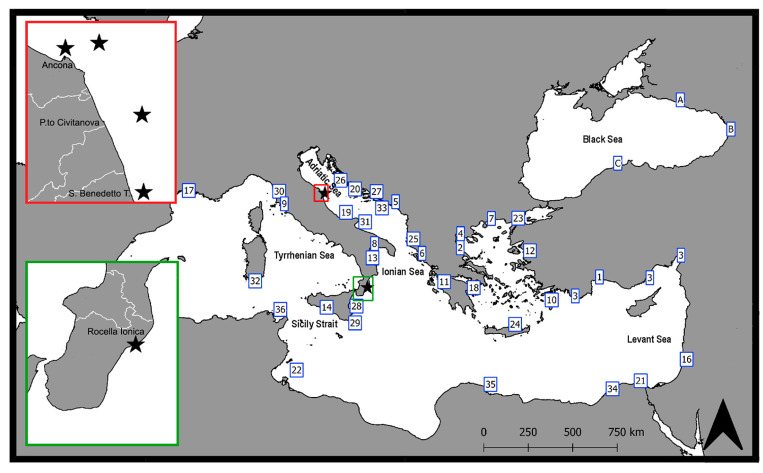
First records of *Penaeus aztecus,* illustrating its spreading in the Mediterranean region. Insets with stars: new records (present study) for west-central Adriatic Sea and Ionian Sea. (See Table 1 for the references of the coded records).

It was reported twice under the latter name from the eastern Black Sea: first in 2006 [14], four years before the first Mediterranean record [7], and again in 2017 [15], well in advance of the “first” record of *P. aztecus* in 2019 off the Turkish Black Sea coast [23]. Khvorov et al. [14] reported the capture in October 2005 of eight specimens of a penaeid shrimp near Bolshoi Sochi, about 150 km from Novorossiysk (the largest commercial Russian port in the Black Sea), and identified them as *P. semisulcatus*. In addition to the description (in Russian), they published photos of the morphological details of the specimens examined that allowed their identification as *P. aztecus*: the carapace in dorsal view shows the adrostral groove and crest extending almost to the posterior margin of the carapace [14] (Figure 3A); the telson has unarmed lateral margins [14] (Figure 3E); pereopod I has two teeth (ischial and basial) and pereopod II only one tooth [14] (Figure 3K).

The second Black Sea report, based on one female caught in September 2014 near the port of Batumi (Georgia) at the extreme East of the Black Sea, about 320 km from the previous locality, was again misidentified as *P. semisulcatus*, but its identity with *P. aztecus* is evident from the photos of the specimen [15] (Figures 2–4, in the latter the images probably became distorted in page composition).

The above misidentifications led to the inclusion of *P. semisulcatus* in the list of alien species in Russian seas [24] (Table 1) and in other reviews of Black Sea decapods fauna [25] and Black Sea NIS [26]. 

In a short note, without shrimp figure or description, Arnesano et al. [16] reported 147 specimens of *P. semisulcatus,* examined in the autumn of 2014 during the monitoring of commercial catches of fishing vessels working in the north-western Ionian Sea (Gulf of Taranto). In a subsequent report of the same monitoring program for the years 2014–2018, Donnaloia et al. [27] cited only *P. aztecus* for the localities referred to in the previous note and added new ones. The finding in 2016 of *P. aztecus* in the Gulf of Corigliano by Renda and Crocetta [28] and the specimens collected in the summer of 2016 in the nearby Roccella Ionica grounds, reported by this study, are pieces of evidence that the report of *P. semisulcatus* in the north-western Ionian Sea [16] was based on a misidentification of *P. aztecus*. The above misidentification [16] led to the inclusion of *P. semisulcatus* in the “New Sightings” of the Italy national report in the ICES WGITMO Report 2016 [29] (p. 105). This error was recognized and corrected in the subsequent ICES WGITMO Report 2017 [30] (p. 74).

### 3.3. Spreading of Penaeus aztecus in the Mediterranean Sea

The literature review suggests that the records from the eastern Black Sea [14,15], reported nine years apart, represent two independent introductions of *P. aztecus*—probably via ballast waters—that did not give origin to any established population.

The first records of *P. aztecus* in the different sectors of the Mediterranean Sea, summarized in Table 1 and mapped in Figure 3, evidence very rapid colonization. Only three years after its first record in 2010 in the Gulf of Antalya [7], a significant population was already established all along the Turkish Mediterranean shelf, from Finike to the Gulf of Iskenderun [8]. By 2013, *P. aztecus* was also recorded in the Thermaikos Gulf (northern Aegean Sea) [9]. The proximity to the port of Thessaloniki, the second commercial port of Greece, again let us guess an introduction of larvae via ballast waters or an unaided expansion of the Turkish population.

The species also very rapidly spread westward, with multiple records since 2015, in the northern part of the Strait of Sicily [31] and in the Tyrrhenian Sea [32]. One specimen was also collected in 2015 in the Gulf of Lion [11], about 60 miles from Marseille, the main French port in the Mediterranean, not followed by additional records. Up to now, this is the westernmost record of the species in the Mediterranean Sea.

The invasion of *P. aztecus* in the southern rim of the Mediterranean Sea shows a similar pattern, with the first capture in 2015 off Israel [11], and the following year off Nile Delta (Egypt) [33] and in the Gulf of Gabes (South Tunisia) [34].

In 2013, captures of single adult specimens of *P. aztecus* were recorded for the first time from the south-eastern side of the Adriatic Sea (Boka Kotorska) [10] and the north-eastern side of the Ionian Sea (off Korfu Island) [35], suggesting an arrival of larvae either carried via ballast waters or drifted by the Levantine current entering in the Adriatic through the Otranto Strait, and flowing northward along the western coast of the Balkan peninsula [36].

The various published reports—all based on one or a few specimens—from different localities in Central Adriatic [12,13] as well as our unpublished records suggest that by 2016, the species was already established in the basin on the eastern and western sides, up to the latitude of 44° N. 

**Table 1:** First records of *Penaeus aztecus* in the Black Sea (letters) and the Mediterranean Sea (numbers), ordered by date. Record codes assigned to the localities as shown in Figure 1. Subsequent records from the same or nearby localities are not listed.

Record Code	Date Collection	Locality	Reference
A	2005	Black Sea: off Lazarevskoe (Russia)	Khvorov et al., 2006 [14] *
B	2014	Black Sea: Batumi (Georgia)	Guchmanidze et al., 2017 [15] *
C	2017	Black Sea: Bozkurt (Turkey)	Gönülal & Türetken, 2019 [23]
1	2009	Levant Sea, Gulf of Antalya	Deval et al., 2010 [7]
2	2012	Aegean Sea: lagunes of Thermaikos Gulf	Nikolopoulou et al., 2013 [9]
3	2012	Levant Sea: Mersin, Finike, Iskenderun	Gökoglu & Özvarol, 2013 [8]
4	2013	Aegean Sea: Thermaikos Gulf	Kevrekidis, 2014 [37]
5	2013	Adriatic Sea East: Boka Kotorska	Marković et al., 2014 [10]
6	2013	Ionian Sea East: Korfu	Kapiris & Apostolidis, 2014 [35]
7	2013	Aegean Sea: Nestos River estuary	Minos et al., 2015 [38]
8	2014	Ionian Sea West: Gulf of Taranto	Arnesano et al., 2015 [16] *
9	2014	Tyrrhenian Sea: Castiglione della Pescaia	Cruscanti et al., 2015 [32]
10	2014	Aegean Sea: off Chalki Island	Kondylatos & Corsini-Foka, 2015 [39]
11	2015	Ionian Sea East: off Kyllini	Zenetos & Giavasi, 2015 [40]
12	2015	Aegean Sea: Çandarlı Bay	Bakir & Aydin, 2016 [41]
13	2015	Ionian Sea West: Gulf of Corigliano	Renda & Crocetta, 2016 [28]
14	2015	Sicily: P.to Empedocle, Mazara del Vallo	Scannella et al., 2017 [31]
15	2015	Ionian Sea West: off Augusta	Donnaloia et al., 2019 [27]
16	2015	Levant Sea: Palmahim	Galil et al., 2017 [11]
17	2015	Gulf of Lion: Le Grau du Roi,	Galil et al., 2017 [11]
18	2016	Aegean Sea: Argolicos Gulf, Vivari lagoon	Kapiris & Minos, 2017 [42]
19	2016	Adriatic Sea West: off Termoli	Zava et al., 2018 [12]
20	2016	Adriatic Sea East: Hvarski kanal	Ugarković & Crocetta, 2021 [13]
21	2016	Egypt: Nile Delta, Damietta	Sadek et al., 2018 [33]
22	2016	Tunisia South: Gulf of Gabes	Ben Jarray et al., 2019 [34]
23	2016	Aegean Sea: Ibrice	Gönülal & Türetken, 2019 [23]
24	2017	Aegean Sea: off Heraklion (Crete Is.)	Kampouris et al., 2018a [43]
25	2018	Adriatic Sea East: Vlora Bay	Kampouris et al., 2018b [44]
26	2018	Adriatic Sea East: Murtersko more	Ugarković & Crocetta, 2021 [13]
27	2018	Adriatic Sea East: Neretvanski kanal	Ugarković & Crocetta, 2021 [13]
28	2018	Ionian Sea West: off Augusta	Pipitone & Lombardo, 2019 [45]
29	2018	Ionian Sea West: off Marzamemi	Kampouris et al., 2018b [44]
30	2018	Ligurian Sea: off Livorno	Ligas et al., 2019 [46]
31	2018	Adriatic Sea West: Gulf of Manfredonia	Donnaloia et al., 2019 [27]
32	2019	Sardinia: off Cape Teulada	Mulas et al., 2019 [47]
33	2019	Adriatic Sea East: off Cavtat	Ugarković & Crocetta, 2021 [13]
34	2019	Egypt: Nile Delta, Abu-Qir	El Deeb et al., 2020 [48]
35	2020	Libya: Gulf of Bomba, Umm-Hufayn lagoon	Abdulrraziq et al., 2021 [49]
36	2020	Tunisia North	Ben Abdallah Ben Hady Hamida et al., 2020 [50]

* Reported as Penaeus semisulcatus.

However, the data of the SoleMon survey (carried out yearly in the North and Central Adriatic Sea from 2016 to 2021 (Table 2)) evidence that it is still very rare compared with the autochthonous species (one specimen of P. aztecus caught in the years 2020 and 2021 versus 1864 and 2124 specimens of P. kerathurus, respectively). 

It is worth noting that in GSA17, the stock of the autochthonous *P. kerathurus* markedly increased in this century [51]. For example, the quantities auctioned in the Ancona gross market rose from 17 tons in 2000 to 82 tons in 2022, with a peak of 95 tons in 2018.

## 4. Discussion

At the collection of a new NIS, it is possible that the species—particularly if native to distant regions—is misidentified, or even described as a new taxon, as in the case of *Lysmata arvoredensis* Giraldes, Macedo, Brandão, Baeza & Freire, 2018. It was described as a new species from the West Atlantic Brasilian coast and later placed in the synonymy of *Lysmata uncicornis* Holthuis & Maurin, 1952, native to the East Atlantic African coast [52]. A species misidentification introduces a “false positive” error in species distribution modelling [53].

Before the appearance of *P. aztecus*, among the penaeid species recorded in the Mediterranean Sea (autochthonous and alien), only *P. semisulcatus* was characterized by the presence of more than one tooth on the lower margin of the rostrum and an unarmed telson. These “distinguishing characters” mentioned in the “CIESM Atlas of exotic species in the Mediterranean—Crustaceans” [54] may have led to the misidentifications, passed unnoticed till now, with the consequent listing of *P. semisulcatus* in the regional lists of NIS [24,25,26] and also in the AquaNIS database [55]. 

*Penaeus semisulcatus* is an earlier Lessepsian immigrant in the Levant Sea, where it is targeted by local fishers since the 1930′s [56,57]. Despite its long-dating presence in the Levant Sea, it did not spread northward into the Aegean Sea [58] (Table 1), [59] (Appendix 1). It is present in the trawling grounds off the Nile Delta [60], but we found no records of its presence westward, except in the species list of an ecological study of a benthic community—which was invaded by the alien alga *Caulerpa cylindracea*—in the Gulf of Salerno (Tyrrhenian Sea) [61] (Appendix). As is often the case with non-taxonomic papers, no vouchered specimens were available to verify whether this report is another misidentification.

In the eastern Mediterranean, the brown shrimp—*P. aztecus*—has proven remarkable invasiveness, quickly becoming of economic value as a fishery resource or as a source of wild fry [33] for the developing shrimp farming industry in Egypt [62]. In other Mediterranean basins, such as the Adriatic Sea, it is still rare. Although it was collected already in 2016 (present records) off Ancona (about at latitude 44° N), it has never been collected further North. The shallow depths and the climatic conditions—winter bottom sea temperatures as low as 10 °C at 30 m depth [63]—are likely to prevent its settlement. The species has not yet been reported from the westernmost part of the Mediterranean (Spain, Algeria, Morocco), where climatic conditions seem favorable, and we may expect its record in the near future. 

Significant longshore movements of *P. aztecus* were reported in the Gulf of Mexico (native area) in a study carried out between 1978 and 1980 [64], with over 71,000 tagged brown shrimps released in different sites of the offshore fishing grounds, and a percentage of recapture of over 12%. Traveled distances of 596 and 528 km from the release point were recorded for two specimens recaptured after 430 and 400 days at sea, respectively. Even considering this capacity of natural dispersion, the multiple records of *P. aztecus*—only 5 years from the first record in the Gulf of Antalya [7]—from sites far away, such as the North Tyrrhenian Sea and the Gulf of Lion, suggest that multiple introduction events have been at the origin of its spreading in the Mediterranean Sea. Unfortunately, the records of *P. aztecus,* with the morphological identification corroborated by molecular data (COI or 16S rRNA sequence) [11], are too scanty to verify any hypothesis. An extensive molecular study of the *P. aztecus* populations through the Mediterranean Sea and the comparison with the genetic sequences available for its American native range may provide insights into the number and origin of introduction events and genetic connectivity among populations, as recently done for another West Atlantic invasive immigrant, the crab *Callinectes sapidus* Rathbun, 1896 [65].

A high number of the NIS species established in the Mediterranean Sea are native to the Indo-West-Pacific region and entered via the Suez Canal, such as *P. semisulcatus*. Others arrived through different introduction pathways from the world oceans. Various studies addressed the role of the different introduction pathways and/or of the native region on the observed distribution of alien species in the Mediterranean region [5,66,67]. Except in the case of intentional introductions of alien species, for which official records may be available, the introduction pathway in the other cases remains an educated/speculative guess. *Penaeus aztecus* is no exception.

In the first record of *P. aztecus* in the Mediterranean Sea, the introduction of larvae via ballast water was suggested as the most likely introduction pathway [7]. Subsequently, other Authors [11,12,13,32] suggested escapes from unreported “clandestine” shrimp farming activities.

The evidence from the history of some shrimp farming attempts in the Mediterranean scarcely support the hypothesis that escape from confinement at aquaculture facilities were the origin of the introduction and spreading of *P. aztecus* in the Mediterranean.

In response to the high demand for penaeid shrimps by the European market, projects to develop shrimp farming in the Mediterranean were launched in the 1970s in various countries such as France [68] and Italy [69]. In the beginning, *P. japonicus* Spence Bate, 1888 was imported from Japan to raise “in loco” the breeding stock necessary to produce shrimp fry. Between 1982 and 1985, about 1.5 million postlarvae (P17–P31) of *P. japonicus* were produced under controlled laboratory conditions by the research center established by the Italian National Research Council in Lesina. These postlarvae were released in spring and harvested in late summer in a “restocking” experiment in the coastal lagoons of Lesina and Varano (Adriatic Sea); small numbers were also intentionally released at sea near lagoon entrances [70]. After these releases, one single adult of *P. japonicus* was caught in the open sea (depth 25 m) in front of the lagoons in December 1985 [71]. A large shrimp hatchery—with a production potential of 6 million postlarvae per year—was established in Sardinia in the 1990s but ceased activity in 2006 [72] before the record of *P. aztecus* in the Mediterranean. A few smaller hatcheries, often inside large fish aquaculture plants, are still present in Italy and other Mediterranean countries, and some cases of unreported “clandestine” import of different species—potential candidates for shrimp aquaculture—have been evidenced [73,74]. The possibility that they also imported *P*. *aztecus* to experiment with its farming cannot be ruled out. 

However, we consider it improbable that aquaculture entrepreneurs in different Mediterranean countries (Turkey, Greece, Italy, and France) almost simultaneously imported *P. aztecus*, with subsequent events of escape from confinement. In addition, the choice of *P. aztecus* seems unlikely as, to our knowledge, it is currently not used in industrial shrimp aquaculture. It was introduced in New Caledonia and French Polynesia (with other alien penaeid shrimps) during the early experiments to develop local shrimp farming [75], but due to its low performance, it was quickly set aside in favor of *P. stylirostris* Stimpson, 1871, which currently accounts for the bulk of production of New Caledonian shrimp farms [76]. 

The earlier record of *P. aztecus* (misidentified as *P. semisulcatus*) in the Black Sea [14], climatically not suited for penaeid shrimp farming, further supports the hypothesis that its presence originated from introductions of larvae/postlarvae via the ballast water of transoceanic vessels departed from the US East coast.

Also worthy of note is the capture in 2018 of a juvenile female (TL 115 mm), tentatively identified with *P. aztecus*, at the mouth of Schelde River (North Sea) near Antwerpen, the main harbor in Belgium [77]. Most likely, it was introduced at the larval stage via ballast water either by a transoceanic vessel departed from the U.S. East Coast, or by a ship of the many lines connecting Mediterranean and North Sea harbors.

Concerns that the presence of *P. aztecus* may “negatively” affect the autochthonous *P. kerathurus* have been expressed [37]; however, at present, no experimental evidence is available. The ecological niche of *P. aztecus* is similar to that of the *Penaeus* species (native or alien) already present in the Mediterranean. Therefore, in areas where a self-sustained population of *P. aztecus* is present, competition for space or food resources seems likely. Local climatology and edaphic conditions, in synergy with the impact of the fishing effort exerted on these highly prized resources, will ultimately determine the abundance of one or another species, probably without substantial changes in ecosystem functioning.

## 5. Conclusions

According to the Marine Strategy Framework Directive, “Non-indigenous species introduced by human activities” is one of the descriptors for determining the good environmental status of marine waters in the EU States [78] and has to be periodically evaluated [79]. Therefore, the correct species identification, hence precise knowledge of its ecological niche in the native region and probable introduction pathways, is fundamental to adopt the more appropriate actions to limit species spreading, keeping in mind that marine spaces are not a backyard and eradication is impracticable once an alien species is established. 

## Figures and Tables

**Table 2:** Abundance of P. kerathurus and P. aztecus in the stations sampled in the North and Central Adriatic (GSA 17) by the fishery survey “SoleMon” in the years 2016–2021.

Year	Sampled Stations N	Stations Positive for *Penaeus* N	*P. kerathurus* N	*P. aztecus* N
2016	74	50	2366	0
2017	70	51	3188	0
2018	68	53	4432	0
2019	68	54	3786	0
2020	58	43	1864	1
2021	63	41	2124	1

## Data Availability

All data used are available in the text.
